# Practical Management in Coronary In-Stent Restenosis: A Narrative Review

**DOI:** 10.3390/jcm15135250

**Published:** 2026-07-05

**Authors:** Handi Y. Salim, Awais Tahir, Wen Hui Teh, Mala Jheinga, Sherab Thaye, Lampson Fan

**Affiliations:** 1 Department of Cardiology, New Cross Hospital, Wolverhampton WV10 0QP, UK; awais.tahir@nhs.net (A.T.); wenhui.teh@uhb.nhs.uk (W.H.T.); mala.jheinga@nhs.net (M.J.); sherab.thaye@nhs.net (S.T.); 2 Department of Cardiology, Birmingham Heartlands Hospital, Birmingham B9 5SS, UK; 3 Department of Cardiology, University Hospital Coventry and Warwickshire, Coventry CV3 2DX, UK; 4 Department of Cardiology, Queen Elizabeth Hospital Birmingham, Birmingham B15 2GW, UK; 5 School of Medicine, University of Birmingham, Birmingham B15 2TT, UK

**Keywords:** coronary in-stent restenosis, intracoronary imaging, percutaneous coronary intervention

## Abstract

Coronary in-stent restenosis (ISR) remains a major contributor to repeat revascularisation despite advances in drug-eluting stent (DES) technology. Its persistence reflects a complex and heterogeneous interplay among mechanical, biological, and procedural factors, and understanding the dominant mechanism in each case is fundamental to effective treatment selection. This narrative review provides a contemporary, mechanism-guided approach to the practical management of coronary ISR. We summarise the definition, incidence, and classification of ISR—including the Mehran, Waksman, and SCAI 2023 time-based frameworks—and outline patient-related, procedural, anatomical, and stent-related risk factors. The pathophysiology of neointimal hyperplasia and neoatherosclerosis is discussed with reference to its clinical implications. Intracoronary imaging with intravascular ultrasound (IVUS) or optical coherence tomography (OCT) is central to ISR characterisation and treatment planning. Current international guidelines support imaging use in ISR management, though it is important to recognise that this recommendation is based largely on observational and surrogate-endpoint data rather than ISR-specific randomised trials demonstrating reductions in hard clinical outcomes, and practical barriers including cost, availability, and operator expertise must be acknowledged. Evidence-based treatment strategies—including drug-coated balloons (DCB), repeat DES implantation, lesion-modifying therapies, vascular brachytherapy, and coronary artery bypass grafting—are reviewed critically with reference to contemporary trial data and their specific clinical applicability. The choice between DCB and repeat DES is addressed with greater nuance, accounting for ISR type (BMS-ISR versus DES-ISR), lesion pattern, stent layering, and bleeding risk. Management considerations in complex subsets—chronic total occlusion ISR, left main ISR, saphenous vein graft ISR, and recurrent ISR—are also addressed. We propose a practical, substrate-driven management framework aligned with the 2024 ESC, 2021 ACC/AHA/SCAI, and 2018 JCS/JSCVS guidelines. Future research priorities include ISR-specific randomised trials with hard clinical endpoints, prospective validation of imaging-guided treatment algorithms, head-to-head comparisons of DCB platforms, and investigation of pharmacological strategies targeting neoatherosclerosis progression.

## 1. Introduction

Despite major advances in drug-eluting stent (DES) technology, coronary in-stent restenosis (ISR) remains a clinically important cause of repeat revascularisation and adverse outcomes [1,2]. In a pooled analysis of 21 randomised trials, ISR was an independent predictor of cardiac mortality with a hazard ratio of 1.23, underscoring that repeat revascularisation for ISR is not a benign event [3].

ISR is mechanistically heterogeneous. Its causes span a spectrum from early mechanical failure—stent underexpansion, malapposition, edge disease, and fracture—to late biological processes including neointimal hyperplasia (NIH), neoatherosclerosis, and drug resistance. This heterogeneity has direct treatment implications: strategies that address one mechanism may be ineffective or even counterproductive in another. A lesion driven by underexpansion requires optimisation of the mechanical substrate before any antiproliferative therapy can succeed; a lesion driven by neoatherosclerosis in a multilayer stent segment may require a fundamentally different approach to one with focal NIH in a well-expanded stent.

Intracoronary imaging with IVUS or OCT plays an important role in identifying the dominant ISR mechanism and informing treatment selection. However, the evidence underpinning this recommendation is largely observational, and the real-world applicability of routine imaging is constrained by availability, cost, operator training, and the absence of ISR-specific randomised controlled trials (RCT) demonstrating improvements in hard clinical outcomes from imaging-guided strategies over angiography-guided care alone. A balanced appraisal of what imaging can and cannot be expected to deliver is therefore important.

Treatment options for ISR have expanded considerably over the past decade, with drug-coated balloons (DCB), repeat DES implantation, lesion-modifying adjuncts, vascular brachytherapy, and surgical revascularisation all having defined but context-dependent roles. The choice between DCB and repeat DES in particular is not binary: outcomes differ meaningfully between BMS-ISR and DES-ISR, between focal and multilayer disease, and between first episode and recurrent ISR. These distinctions are not consistently reflected in practice.

This narrative review synthesises contemporary evidence on ISR incidence, pathophysiology, diagnosis, and management with a mechanism-guided, substrate-based approach. Where evidence is strong and consistent, we say so; where it is limited, observational, or based on surrogate endpoints, we identify those limitations explicitly. The aim is to provide a practically useful framework for clinical decision-making that is grounded in a critical reading of the available evidence rather than a summary of it.

## 2. Methods

This narrative review was conducted following a systematic search of PubMed and MEDLINE databases for articles published between January 2000 and March 2026. Search terms included “in-stent restenosis,” “coronary restenosis,” “drug-eluting stent restenosis,” “drug-coated balloon,” “intravascular ultrasound ISR,” “optical coherence tomography ISR,” “neointimal hyperplasia,” “neoatherosclerosis,” “vascular brachytherapy,” and “stent fracture,” used individually and in combination. RCTs, meta-analyses, large registry studies, prospective observational studies, and major international guidelines from the ESC, ACC/AHA/SCAI, and JCS/JSCVS were prioritised. Studies were selected based on their clinical relevance, sample size, methodological rigour, and publication date, with emphasis on contemporary evidence from the last decade. Key landmark trials and seminal mechanistic studies from earlier periods were also included where relevant.

## 3. Definition and Incidence of In-Stent Restenosis

The Academic Research Consortium (ARC-2) defines ISR as the re-narrowing of a coronary artery lumen within the stented segment and/or within 5 mm segments proximal and distal to the stent, resulting in ≥50% luminal diameter stenosis, or >75% of the reference vessel cross-sectional area reduction on intravascular imaging [4,5].

Clinically relevant ISR should be confirmed by recurrent ischaemic symptoms, objective evidence of ischaemia (e.g., ECG or perfusion testing), abnormal physiological indices with fractional flow reserve (FFR ≤ 0.80) or instant wave-free ratio (iFR ≤ 0.89), or angiographic stenosis > 70% in asymptomatic patients [6].

**Figure 1:**
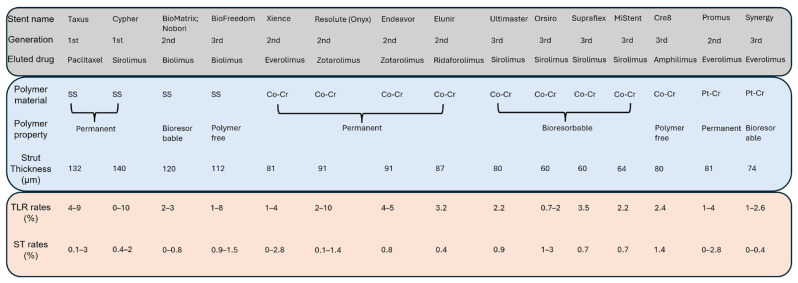
Evolution of drug-eluting stent platforms and associated rates of target lesion revascularisation (TLR) and stent thrombosis (ST) at one year. Co, cobalt; Cr, chromium; Pt, platinum; SS, stainless steel; TLR, target lesion revascularisation; ST, stent thrombosis. Adapted from Giustino et al. [8].

Contemporary DES platforms demonstrate an annual risk of ISR of approximately 2%, though this figure varies considerably according to patient-related risk factors, lesion complexity (small vessel, long lesion, bifurcation), stent generation, and duration of follow-up, with rates rising substantially in higher-risk subsets (Figure 1) [7].

## 4. Classifications of ISR

Structured classification provides a framework for mechanism-based therapy. Although a single universal system is lacking, three major classification systems are commonly used: Mehran, Waksman, and The Society for Cardiovascular Angiography and Interventions (SCAI) 2023 time-based classification.

### 4.1. Mehran Classification

Originally developed for bare metal stent (BMS) ISR, this angiographic scheme delineates four lesion patterns [9]. The classification includes: Type I (Focal), lesions ≤ 10 mm within the stent; Type II (Diffuse, intra-stent), lesions > 10 mm within the stent; Type III (Diffuse, proliferative), >10 mm extending beyond stent edges; and Type IV (Occlusive), total stent occlusion.

Focal lesion (Type I) is likely to respond favourably to balloon angioplasty (BA) and DCB, while more complex lesions (Type III and IV) often require adjunctive modifications before DCB or DES treatment, or even surgical revascularisation.

### 4.2. Waksman Classification

The Waksman classification, emphasising intravascular imaging for ISR mechanism identification and treatment guidance, categorises ISR as: Type I (Mechanical), due to stent underexpansion or fracture; Type II (Biological), driven by NIH or neoatherosclerosis; Type III (Mixed), a combination of mechanical and biological factors; and Type IV, chronic total occlusion [10].

The Waksman classification guides the management approach based on the mechanism of ISR. Mechanically driven ISR (Type I) may respond well to lesion optimisation with high-pressure BA and DCB as a first-line treatment, whereas biologically driven ISR (Type II) tends to be more diffuse and prone to recurrence, often requiring antiproliferative therapy (DES or DCB). Mixed and occlusive types carry worse long-term outcomes and may require adjunctive lesion modification, such as atheroablative therapy or intravascular lithotripsy (IVL).

### 4.3. SCAI 2023 Time-Based Classification

This classification describes ISR presentation relative to the time following index PCI. Early ISR (≤30 days post-PCI) suggests technical or procedural cause in origin (e.g., underexpansion, malapposition), whereas late ISR (30 days to 1 year post-PCI) and very late ISR (>1 year post-PCI), often reflect biological processes such as NIH or neoatherosclerosis [11].

## 5. Risk Factors for ISR

ISR may be attributed to a confluence of factors encompassing patient-specific characteristics, procedural techniques, anatomical considerations, and stent-related properties (Table 1).

### 5.1. Patient-Related Factors

Older age, hypertension, diabetes mellitus, dyslipidaemia, chronic kidney disease, smoking, and a previous history of coronary artery bypass grafting (CABG) have been identified as independent predictors of ISR [8]. Diabetes mellitus, through hyperglycaemia or hyperinsulinaemia-induced metabolic and inflammatory disturbances, accelerates NIH formation, impairs endothelial healing and promotes adverse vascular remodelling [6]. A study by Mone et al. demonstrated that hyperglycaemia increases the risk of ISR ~2–3 fold at one-year follow-up in STEMI patients, regardless of stent type [12].

Dyslipidaemia similarly predisposes to ISR by damaging vascular endothelial cells, accelerating atherosclerotic plaque formation and facilitating smooth muscle proliferation within the stent. Elevated lipoprotein(a) (≥30–50 mg/dL) is associated with ~1.5 fold increased risk of ISR [13].

Biological mechanisms also play a key role. Drug resistance may be primary (genetically predisposed) or secondary (following drug exposure), and hypersensitivity or inflammatory reactions to stent metals or polymer coatings promote NIH [6].

Stent implantation induces local vascular injury and inflammation which potentially delays arterial healing and stent endothelialisation [14]. NIH, a key mechanism in ISR, is initiated by vascular injury during stent implantation and accelerated by local and systemic inflammatory responses [15].

### 5.2. Procedural Factors

Suboptimal stent deployment, including malapposition and underexpansion, increases ISR risk. Acute stent malapposition occurs often due to stent underexpansion or challenging anatomy (e.g., calcification, large vessel diameters) [16]. Late stent malapposition, potentially due to positive vascular remodelling, occurs more frequently with DES than with BMS. While acute malapposition is not associated with increased stent-related adverse events, late malapposition correlates with increased risk of DES failure, including ISR and thrombosis [17].

Stent underexpansion and suboptimal post-procedural minimal stent area (MSA) are strongly associated with decreased long-term DES patency, increased risk of ISR and stent thrombosis, particularly within the first year post-implantation [18]. Stent underexpansion can be mitigated by using intravascular imaging and avoiding MSA threshold predictive of ISR (e.g., intravascular ultrasound (IVUS) MSA < 5.5 mm^2^ in non-left main (LM) vessels, <6.0 mm^2^ in LM; optical coherence tomography (OCT) MSA < 4.5 mm^2^) [19,20,21]. While stent underexpansion is primarily caused by suboptimal lesion preparation, inadequate sizing or stent post-dilation, it can also result from stent recoil, a factor implicated in ISR development [22].

### 5.3. Anatomical Factors

Vessel size, lesion morphology, and local haemodynamics significantly influence ISR. A meta-analysis showed that small vessels, longer lesions and longer stents increased the risk of ISR [23]. A stent in a lesion > 20 mm long poses a higher risk of restenosis than a shorter lesion [24]. In addition, stents in lesion type B2/C, bifurcation lesion, ostial lesion and smaller vessel size are associated with increased risk of ISR [23,25,26]. Stenting in smaller vessels (<3 mm) has been shown to have a 20% restenosis rate due to their reduced post-procedural MSA, a higher degree of vessel injury and recoil, and increased metallic density within the vessel wall [26,27,28]. A prospective study directly comparing DES in small (<3 mm) and large (≥3 mm) vessels in the same patients found six-month restenosis rates of 24% in small vessels versus 15% in large vessels [28]. These factors collectively exacerbate NIH and compromise long-term stent patency.

Lesion characteristics, including high thrombus burden and severe calcifications, further contribute to ISR. Thrombus impedes antiproliferative drug distribution and delayed resolution can lead to malapposition, adverse remodelling, and stent failure [29].

Calcification increases the incidence of ISR by several mechanisms, including suboptimal stent expansion, increased NIH, and the development of calcified neoatherosclerosis or calcified nodules within the stent. The presence of large arcs (>180°) or thick calcium (>0.5 mm) within or adjacent to the stent is associated with five times higher rates of ISR [30,31]. Calcified neoatherosclerosis is a recognised substrate for very late ISR; its prevalence increases over time following stent implantation and it is associated with poor results after reintervention [32].

PCI of coronary bifurcations, compared to non-bifurcation lesions, exhibits higher ISR rates, with the stenting technique significantly influencing outcomes [8]. ISR in bifurcation PCI is frequently observed at the side branch ostium and carina, and is associated with wider bifurcation angle [33,34].

For most bifurcation lesions, provisional stenting remains the default approach due to its procedural simplicity and comparable outcomes to two-stent strategies [35]. However, further studies and meta-analysis demonstrated that, particularly in LM bifurcation, the double-kissing crush (DK crush) technique has been shown to yield lower rates of ISR and major adverse cardiovascular events (MACE) [35,36,37,38].

### 5.4. Stent-Related Factors

Stent-related factors contributing to ISR include stent type, drug distribution and properties, strut thickness, and stent fracture.

The advances in contemporary DES have arisen from the optimisation of anti-proliferative agents, the use of more bio-compatible polymers or polymer-free material, and the reduction in stent strut thickness (Figure 1). The timeline of neointimal accumulation differs between BMS and DES. BMS exhibits peak late lumen loss at 6–8 months, followed by a decline, whereas DES demonstrates gradual accumulation persisting for up to 5 years [1]. Notably, neoatherosclerosis occurs more frequently and earlier in DES compared to BMS [39].

Ultrathin stent struts (<70 μm) have been shown to reduce target lesion failure by 16% at one year in a meta-analysis comparing ultrathin-strut DES to second-generation DES counterparts [40]. Furthermore, the reduction was maintained in a subsequent meta-analysis at longer median follow-up of 2.5 years [41]. However, the benefits of thinner struts are not universal. In chronic total occlusions (CTOs), ultrathin-strut (~60 μm) sirolimus-eluting stents have shown increased late lumen loss compared to thin-strut (~81 μm) everolimus-eluting stents [42].

Stent gaps, characterised by discontinuous lesion coverage between adjacent stents, increase ISR and repeat revascularisation risk [43]. In DES, these gaps lack antiproliferative drug exposure and mechanical support.

Stent fracture is defined as a complete or partial disruption of the structural integrity of a stent that was initially continuous following implantation. In DES, stent fracture compromises local drug delivery and mechanical support. Risk factors include implantation in the right coronary artery, vessel tortuosity, and long or overlapping stents [1,44]. Stent fracture incidence ranges from 1 to 8%, with revascularisation rates of 15–60% [44].

## 6. Pathophysiology of ISR

Inflammation plays a pivotal role in ISR. Following PCI, endothelial cells are activated by pro-inflammatory cytokines [45]. These pro-inflammatory cytokines stimulate growth factors, leading to the activation, migration, and proliferation of vascular smooth muscle cells (VSMCs), initiating the restenotic process. Clinically, this mechanistic insight underpins the rationale for antiproliferative agents in DES and DCB, which locally suppress VSMC proliferation and inflammation, mitigating early NIH [46,47].

NIH, a key ISR mechanism, involves smooth muscle cell accumulation and extracellular matrix deposition within the intima. This process is characterised by vascular smooth muscle cell proliferation and migration from the media to the intima, accompanied by extracellular matrix production [1]. Early NIH explains the efficacy of the antiproliferative agents in DES and DCB targeting this hyperplasic pathway and mitigating restenosis [47].

Neoatherosclerosis constitutes another significant biological mechanism contributing to ISR, particularly in cases occurring more than one year after DES implantation [39]. It is characterised by an accumulation of lipid-laden macrophages, with or without a necrotic core, and/or calcifications within the neointima. A study by Nakazawa et al. found that the anti-proliferative drugs in DES resulted in delayed endothelial healing [48]. Hence, while DESs are effective in reducing early NIH, they can be associated with late neoatherosclerosis, which is a common finding in recurrent DES-ISR. Understanding ISR mechanisms and pathophysiology is important as contemporary ISR management is increasingly substrate-driven and imaging-guided.

## 7. Diagnosing ISR

Various imaging modalities are available for detecting ISR. Each modality offers distinct strengths and limitations in the diagnosis of ISR.

### 7.1. Coronary Angiography

Coronary angiography remains the initial reference test for assessing ISR, but its two-dimensional nature limits lesion characterisation, particularly in complex anatomy, overlapping stents, diffuse disease, and eccentric NIH [5,49]. Visual estimation is also subject to projection dependence and inter-observer variability.

Haemodynamic assessment with positive functional assessment (FFR ≤ 0.80 or iFR ≤ 0.89) can help determine whether reintervention is warranted where the angiographic severity is moderate or the clinical significance is uncertain [6].

### 7.2. Intravascular Ultrasound (IVUS)

IVUS provides visualisation of the external elastic lamina for optimal stent expansion and evaluates ISR substrates, such as stent underexpansion, fracture, and NIH [50,51]. IVUS also aids in accurate vessel sizing, but its lower axial resolution (~150 μm) limits fine neointimal tissue characterisation [51]. IVUS-derived (MSA) thresholds—<5.5 mm^2^ in non-LM vessels, <6.0 mm^2^ in LM—have been consistently associated with ISR risk in observational and registry data [19,20,52,53,54]. Thus, achieving MSA ≥ 5.5 mm^2^—with further optimal MSA of 6 mm^2^ for the LAD ostium, 7 mm^2^ for the LM/LAD/LCx bifurcation, and 8 mm^2^ for LM PCI—is a reasonable procedural goal [23,55]. A relative stent expansion of 80% is favourable, with 90% being ideal [56]. High plaque burden at stent edges and edge dissection greater than 60^o^ or 3 mm in length are risk-predictors for ISR [57].

### 7.3. Optical Coherence Tomography (OCT)

**Figure 2:**
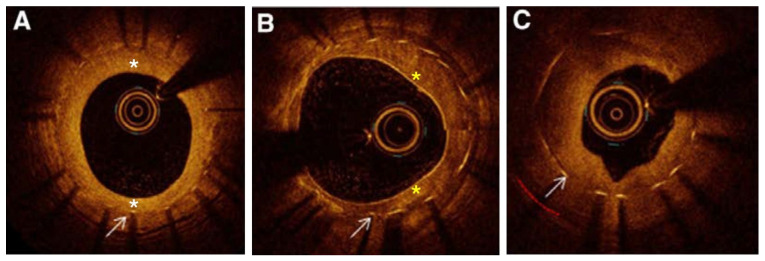
Optical coherence tomography (OCT) images of in-stent restenosis. Stent struts are indicated with white arrows in each panel. Panel (**A**) shows a bright, homogeneous layer of neointimal hyperplasia (white asterisks). Panel (**B**) shows a heterogeneous in-stent composition with a necrotic core and thin calcified plaque cap (yellow asterisks). Panel (**C**) shows stent underexpansion, with stent struts distant from the external elastic lamina (red line) and heterogeneous intimal hyperplasia. Reproduced with permission from CRTOnline.org.

OCT provides higher-resolution cross-sectional imaging (~15 µm axial resolution) with superior neointimal tissue characterisation, enabling detection of NIH composition, neoatherosclerotic features (lipid infiltration, macrophage accumulation, calcified plaques, necrotic core), thrombus, tissue protrusions, and edge dissections (Figure 2) [51,58]. Its major limitation is reduced tissue penetration compared with IVUS, which restricts assessment of the vessel wall beyond the stent. OCT-derived MSA < 4.5 mm^2^ and MSA/reference lumen area < 70% are associated with higher ISR risk, and the presence of heterogeneous neointima, in-stent neoatherosclerosis, or calcification can identify the biological substrate driving recurrence [59,60,61].

### 7.4. IVUS or OCT: A Practical Framework

IVUS and OCT are complementary rather than competing tools (Table 2). IVUS is often preferable when the issue is vessel sizing, deep plaque burden, or stent underexpansion in calcified disease, whereas OCT is often favourable when fine structural detail, thrombus, edge dissection, or neoatherosclerosis is the primary concern.

**Figure 3:**
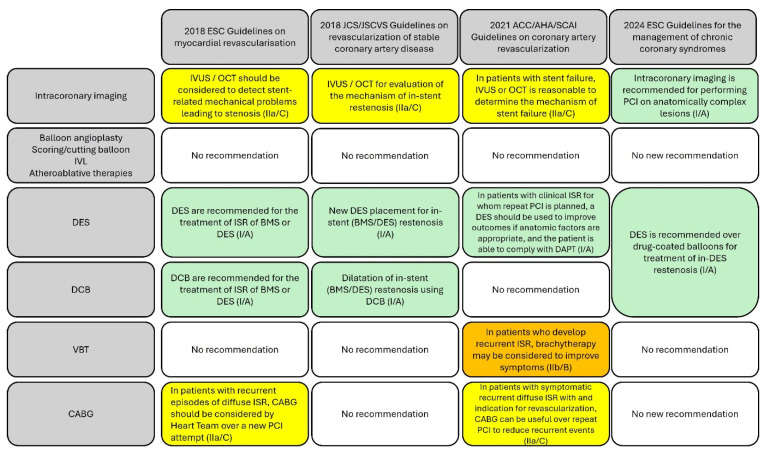
Guidelines recommendations for the management of ISR. This figure summarises the guidelines from the ESC, ACC/AHA/SCAI, and JCS/JSCVS regarding ISR management. Common key recommendations include the use of intravascular imaging, and support the use of DCB and DES. (I/A), class I recommendation and level of evidence A. Multiple class/level combinations are depicted in this figure. The respective colours represent the class recommendation; Green, class I recommendation; Yellow, class IIa recommendation; Orange, class IIb recommendation; ACC, American College of Cardiology; AHA, American Heart Association; BMS, bare-metal stent; CABG, coronary artery bypass graft; DCB, drug-coated balloons; DES, drug-eluting stent; ISR, in-stent restenosis; IVL, intravascular lithotripsy; IVUS, intravascular ultrasound; JCS, Japan Circulation Society; JSCVS, Japanese Society for Cardiovascular Surgery, OCT, optical coherence tomography; PCI, percutaneous coronary intervention; SCAI, Society for Cardiovascular Angiography and Interventions; VBT, vascular brachytherapy.

Either modality can improve mechanistic understanding, and major international guidelines recommend the use of intracoronary imaging when feasible (Figure 3) [62,63,64,65]. The 2024 ESC guidelines assign IVUS or OCT-guided PCI a Class I, Level B recommendation for ISR assessment and treatment optimisation. The 2021 ACC/AHA/SCAI guidelines similarly support intracoronary imaging use in ISR with a Class IIa, Level B recommendation.

However, it is important to acknowledge that the evidence underpinning these recommendations is derived largely from observational registries, post hoc imaging substudies, and surrogate endpoint trials rather than from ISR-specific RCT demonstrating improvements in hard clinical outcomes such as death or myocardial infarction. Practical barriers—including equipment cost, contrast requirements, training and operator experience, and procedure time—limit availability across different healthcare settings.

Imaging should therefore be recommended as the standard of care in centres with appropriate expertise, while acknowledging that not all ISR presentations in all settings will routinely access it.

## 8. Management of ISR

**Figure 4:**
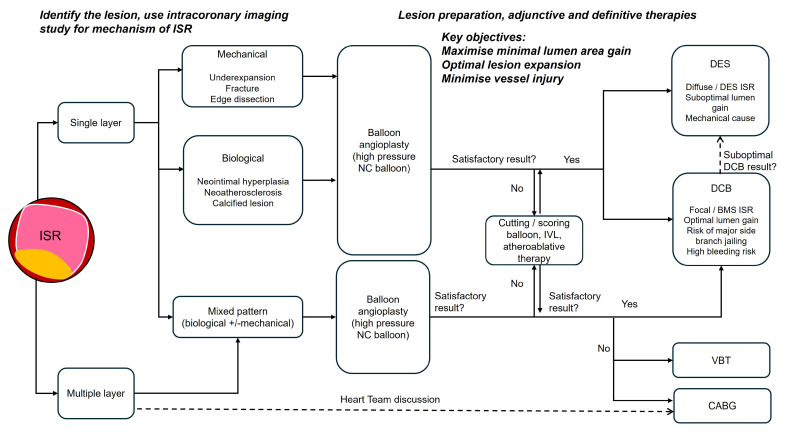
Central illustration step-wise algorithm management for ISR. BMS, bare-metal stent; NC, non-compliant; IVL, intravascular lithotripsy; DES, drug-eluting stent; DCB, drug-coated balloons; VBT, vascular brachytherapy; CABG, coronary artery bypass graft.

The guideline recommendations and step-wise approach for ISR management are summarised in Figure 3 and Figure 4, respectively. The central principle is that treatment should follow from the identified mechanism: lesion preparation addresses the immediate substrate, and definitive therapy should match the dominant phenotype—mechanical, biological, or mixed.

### 8.1. Balloon Angioplasty (BA)

Plain BA carries a high recurrence rate when used as stand-alone therapy and should generally be regarded as a lesion preparation step rather than a definitive treatment [66]. It remains useful when the primary problem is underexpansion, particularly when high-pressure, non-compliant balloons are needed, but further lesion modification or definitive therapy is usually required.

### 8.2. Cutting and Scoring Balloons

Cutting and scoring balloons can improve lesion preparation in fibrotic, resistant, or mildly calcified ISR, especially when standard balloon inflation fails to achieve adequate expansion. Their value is largely procedural: they improve drug-delivery geometry and facilitate subsequent DCB or DES therapy rather than independently providing durable antiproliferative benefit.

The ISAR-DESIRE 4 trial supports this preparation-first concept by showing better angiographic outcomes when scoring balloon preparation preceded DCB treatment [67]. Specifically, it was an RCT comparing neointimal modification with scoring balloon pre-dilation before DCB versus standard DCB therapy. The trial demonstrated that scoring balloon pre-dilation improved the anti-restenotic efficacy of DCB therapy, resulting in lower rates of angiographic restenosis and percent diameter stenosis at follow-up.

The RESCUT trial also suggested improved balloon deliverability and less slippage, although it did not demonstrate a clear reduction in recurrent ISR [68]. These data support the selective use of scoring and cutting balloons as lesion preparation adjuncts, not as definitive therapies in their own right.

### 8.3. Intravascular Lithotripsy (IVL)

IVL modifies calcified plaque using pulsatile sonic pressure waves and may be particularly useful in ISR where calcium-mediated underexpansion is the dominant mechanical problem and where conventional balloon inflation or atherectomy carries an unacceptable risk of perforation or vessel trauma [69]. Current evidence in ISR is limited to small series and selected case reports; IVL should therefore be positioned as an adjunctive strategy in anatomically appropriate cases.

### 8.4. Atheroablative Therapy

Atheroablative therapy debulks coronary plaque, ablates calcified tissue and facilitates greater luminal gain. Rotational atherectomy (RA) and excimer laser coronary angioplasty (ELCA) are the two most commonly used atheroablative techniques in this setting.

RA may be considered when dealing with an arc of calcium > 270° and >0.67 mm thickness. RA can be used when stent underexpansion is due to >90° peri-stent calcium. While the RA burr can ablate metallic stent struts, its use in underexpanded stents necessitates caution to prevent burr entrapment. The evidence remains limited whether routine use of RA improves clinical outcomes compared with BA alone [70].

ELCA utilises an excimer laser to generate monochromatic light energy, which is absorbed by plaque, resulting in plaque disruption through heat and shock waves. An observational study found that ELCA effectively ablates NIH and was associated with a high procedural success rate and a low incidence of periprocedural complications compared with BA alone [71]. ELCA should be used cautiously in underexpanded stents due to the risk of vessel perforation and severe no-reflow.

Orbital atherectomy (OA), another device approved for use in de novo severely calcified coronary lesions, employs an elliptical burr mechanism to achieve atheroablation. However, experience with OA for ISR treatment is limited to a single-centre, retrospective observational study [72]. Thus, the use of OA in ISR is limited at present.

The role of atheroablative therapies is mainly to facilitate lesion modification before definitive therapy. However, the evidence base is sparse, largely non-randomised, and highly selected, so these techniques should be described as niche adjuncts rather than routine ISR treatments.

### 8.5. DCB vs. Repeat DES

The 2018 ESC, 2018 JCS/JSCVS and 2021 ACC/AHA/SCAI guidelines recommend DCB or DES as a Class Ia indication to treat ISR [62,63,64]. Notably, the recent 2024 ESC guidelines shift their recommendation towards DES over DCB specifically for DES-ISR treatment (Class Ia indication) [65]. This nuance matters: the two strategies are not equivalent in all ISR presentations.

#### 8.5.1. BMS-ISR vs. DES-ISR

The DAEDALUS study, an individual patient data meta-analysis of 10 RCTs including 1976 patients, found that DCB and DES have similar efficacy and safety in BMS-ISR at three years. In DES-ISR, however, DES was found to be moderately more effective than DCB in reducing TLR at three years, with no differences in mortality looking at repeat DES versus paclitaxel-DCB [73]. This distinction is clinically important. In BMS-ISR, where the ISR substrate is predominantly biological NIH without prior antiproliferative drug exposure, DCB represents an equally effective and metal-free option. In DES-ISR, particularly recurrent or multilayer disease, repeat DES may confer greater antiproliferative efficacy, though at the cost of adding further metallic layers.

#### 8.5.2. Lesion Complexity and Multilayer Stenting

In long or diffuse ISR segments, repeat DES increases cumulative metal burden, which is itself a risk factor for future restenosis and complicates subsequent revascularisation. DCB avoids this concern and is particularly advantageous in multilayer restenotic segments, ostial or bifurcation ISR where side branch jailing is a concern, and in patients where future bypass grafting may be required.

In a study of two large registries (DEB-DRAGON and ULTRA), ultrathin-strut DES were associated with reduced TLR and TVR in ISR compared with thin-strut DES and DCB at three-year follow-up, suggesting a role for contemporary DES platforms when repeat DES is chosen, though these are observational comparisons [74].

#### 8.5.3. Long-Term Outcomes and Evidence Gaps

A meta-analysis by Maqsood et al. found no difference in MACE and TLR between DCB and DES as primary treatment for ISR [66]. However, long-term data beyond three years for DCB remain limited and represent an important evidence gap. The SELUTION4ISR trial demonstrated non-inferiority of sirolimus-coated balloon to standard of care at one year, with longer-term follow-up ongoing [75]. Recent studies and registries suggest comparable clinical efficacy and safety between paclitaxel and sirolimus-based DCB in both de novo and ISR setting, though head-to-head RCT data remain sparse [76,77].

#### 8.5.4. Regional and Practical Considerations

Practice patterns for DCB versus DES in ISR vary considerably across regions, reflecting differences in device availability, reimbursement policies, and operator familiarity. In Europe and the UK, DCB use in ISR is well established and guideline-supported with the UK regulatory body supporting DCB cost-effectiveness [78].

In North America, uptake has historically been more limited, partly reflecting slower regulatory approval of DCB platforms. However, the trend is changing with the first paclitaxel-based DCB approval for coronary ISR, and the registry data show growth in DCB use in ISR PCI [79].

#### 8.5.5. Practical Decision Framework

In practice, the choice between DCB and repeat DES should be individualised. DCB avoids an additional metal layer and is particularly attractive in: long or diffuse ISR where further scaffolding would increase metallic density; multilayer ISR where each additional stent layer reduces future expandability and drug penetration; cases where side branch jailing is a concern; and high bleeding-risk patients who would benefit from a shorter DAPT course. Repeat DES provides more reliable acute luminal gain and scaffold support, and is preferred when: mechanical failure requires correction that cannot be achieved by balloon preparation alone; the original underexpansion has not been resolved; or prior DCB treatment has already failed [80]. Operator experience and local availability legitimately influence this decision in real-world practice, and regional variation in DCB use should be acknowledged.

### 8.6. Vascular Brachytherapy (VBT)

VBT delivers localised beta-radiation to inhibit NIH without adding a metallic implant, making it conceptually attractive in multilayer DES-ISR where additional stent layers are undesirable. A study by Tanner et al. showed that VBT strategy in multilayer DES-ISR was associated with lower MACE compared with non-VBT strategy at three-year follow-up [81]. VBT carries a Class IIb recommendation in the ACC/AHA guidelines and SCAI consensus, reflecting modest and non-randomised evidence and limited availability [11,62]. It should be reserved for recurrent or multilayer ISR after failure of conventional therapy, in centres with appropriate infrastructure.

### 8.7. Coronary Artery Bypass Graft (CABG)

CABG can be useful for selected patients with LM-ISR, multivessel disease, recurrent ISR, or ostial LAD involvement with a Class IIa recommendation in both American and European guidelines [62,63,65]. The decision should be individualised and ideally discussed within a Heart Team framework, especially when repeated percutaneous strategies are unlikely to provide durable benefit.

A study by Gaudino et al. showed that CABG can achieve durable revascularisation with arterial grafts showing higher patency rates and better long-term outcomes compared to saphenous vein graft (SVG) grafts in ISR patients [82]. However, when compared to grafting native vessel, grafting to ISR vessel is associated with an increased risk of TVR, highlighting the need for careful patient selection [83].

### 8.8. Pharmacological Therapies and Cardiovascular Risk Factor Modifications

Pharmacological therapy is an integral component of ISR management, targeting residual thrombotic risk, NIH, and systemic atherosclerosis progression.

#### 8.8.1. DAPT

DAPT duration following PCI for ISR should be individualised according to the clinical presentation (stable or acute coronary syndrome), the device used (DCB or repeat DES), and the patient’s bleeding and ischaemic risk profile, rather than applying a single default recommendation to all patients.

For patients treated with repeat DES, the 2024 ESC and 2021 ACC/AHA/SCAI guidelines support a minimum of one month of DAPT regardless of indication, with extension to six or twelve months in most patients depending on ischaemic risk, ACS presentation, and tolerability [62,63,65]. This is consistent with guidance for de novo DES implantation and reflects the thrombotic risk of the stented segment rather than ISR-specific evidence, since no large RCT has specifically evaluated DAPT duration after repeat DES for ISR.

For patients treated with DCB without additional stent implantation, DAPT can generally be shortened. The ESC guidelines suggest one month of DAPT after DCB-only treatment in stable patients, which may be preferable in those with high bleeding risk. This shorter duration is supported by the absence of a permanent metallic implant and the transient pharmacological effect of the balloon, though the optimal minimum duration remains uncertain in the absence of large ISR-specific randomised evidence.

In patients presenting with ACS-related ISR, a longer DAPT course is appropriate regardless of device, consistent with guideline recommendations for ACS in general. A subgroup analysis of the PRODIGY trial found that prolonged DAPT was associated with a significant reduction in MACE at 24 months (7.3% vs. 16.7%) among patients who underwent PCI for ISR, suggesting a potential ischaemic benefit from extended therapy in this subset [84]. However, this was a post hoc subgroup analysis of a trial not designed specifically for ISR, and its findings cannot yet be translated into a firm recommendation without prospective replication. The benefit of prolonged DAPT must be weighed against the increased risk of bleeding. Clinical decision-making tools such as the PRECISE-DAPT and ARC-HBR scores can assist in identifying patients at high bleeding risk in whom shorter DAPT courses may be preferable.

In patients at high bleeding risk—including those on oral anticoagulation, the elderly, or those with recent bleeding—DAPT should be minimised in duration, and DCB-based treatment may be preferred when technically appropriate. The decision should also account for whether the patient is on long-term anticoagulation, which would generally favour the shortest effective DAPT course possible.

#### 8.8.2. Lipid-Lowering Therapy

Intensive lipid-lowering therapies are reasonable given the role of neoatherosclerosis in late ISR. Current ACC/AHA guidelines recommend high-intensity statin therapy for secondary prevention in patients with established CAD, and additional PCSK9 inhibition may be warranted in patients who remain above LDL targets despite maximally tolerated statin therapy [62,85].

There is no direct randomised evidence demonstrating that intensive lipid-lowering reduces ISR rates on angiographic endpoints, though the PIECES-OCT trial is evaluating whether early PCSK9 inhibition improves stent strut endothelial coverage as a surrogate marker [86]. The primary endpoint is OCT-assessed stent strut endothelial coverage at 12 weeks—a surrogate marker—and the trial is designed to assess whether early PCSK9 inhibitor initiation can improve strut coverage and potentially reduce ISR rates.

#### 8.8.3. Other Cardiovascular Risk Factors Optimisation

Optimisation of modifiable risk factors—blood pressure control, glycaemic management in diabetes, smoking cessation, and physical activity—is part of secondary prevention following any coronary revascularisation. In ISR specifically, poor glycaemic control and elevated lipoprotein(a) have been associated with increased recurrence risk, reinforcing the importance of medical optimisation alongside mechanical intervention.

## 9. Management of ISR in Special Situations

### 9.1. Chronic Total Occlusion in ISR (CTO-ISR)

CTO-ISR represents one of the most technically demanding subsets of ISR. It accounts for approximately 10–15% of all CTOs treated with PCI and occurs in a patient population with a markedly higher burden of comorbidity than de novo CTO—including higher rates of diabetes, multivessel disease, and prior CABG—which itself predicts worse outcomes independent of procedure success [87].

A pooled analysis reported comparable technical and procedural success rates (approximately 85%) between CTO-ISR and de novo CTO, suggesting that experienced operators can achieve acceptable procedural outcomes [87]. However, 12-month MACE rates are higher in CTO-ISR than in de novo CTO, likely reflecting both the underlying patient complexity and the biological challenges of treating a recurrent occlusion within a previously stented segment. Whether this elevated MACE reflects procedural limitations, disease progression, or patient selection bias is difficult to disentangle from the available observational data.

Pragmatically, CTO-ISR should be approached by operators with dedicated CTO experience. Pre-procedural intracoronary imaging—where feasible—can clarify the mechanism of occlusion and guide wire strategy. The choice between DCB and repeat DES following successful recanalisation should follow the same substrate-driven framework applied in non-occlusive ISR, with repeat DES generally preferred when the mechanistic problem requires scaffolding correction.

### 9.2. LM-ISR

LM-ISR carries particular prognostic weight. Observational data from the EXCEL long-term follow-up have shown that repeat LM revascularisation, whether percutaneous or surgical, is independently associated with increased all-cause and cardiovascular mortality [88]. This finding underlines the importance of preventing LM-ISR through optimal primary technique, but also of making careful treatment decisions when it occurs.

When LM-ISR is identified, the mechanism should be defined with intracoronary imaging before reintervention. Underexpansion of the LM stent—particularly at the ostium or bifurcation—is a common mechanical substrate and, if correctable, may respond well to optimisation with high-pressure balloons and repeat DES. Biological ISR in the LM setting, especially when diffuse or neoatherosclerotic, carries higher recurrence risk with any percutaneous strategy.

Observational data suggest DES and DCB can achieve comparable angiographic results in LM-ISR, but these studies are small and non-randomised [89,90]. Surgical revascularisation should be considered in preference to repeat PCI when anatomy is suitable and operative risk is acceptable, particularly in the setting of recurrent or complex LM-ISR. Consistent with this, the 2024 ESC and 2021 ACC/AHA/SCAI guidelines assign CABG a Class IIa recommendation for this context. The decision should be made by the Heart Team, weighing the long-term durability of surgery against the procedural risk [62,65].

### 9.3. SVG-ISR

PCI to SVG-ISR is technically challenging and carries risk that differs substantially from native-vessel ISR. Vessel friability, superimposed thrombus, and vulnerability to distal embolisation and no-reflow make outcomes less predictable than in native-vessel ISR [91,92].

The evidence base is sparse: the DIVA trial evaluated DES versus BMS for de novo SVG PCI (not specifically ISR) and found no significant difference in clinical outcomes between stent types, though it is unclear how directly applicable these findings are to the SVG-ISR subset specifically [93,94]. The PROCTOR trial demonstrated lower MACE at one year with SVG PCI compared with native-vessel PCI (19% vs. 34%) in some scenarios, but again this was not an ISR-specific study [95]. As a result, the current evidence for PCI to SVG-ISR is largely observational and limited.

When feasible, native-vessel revascularisation—bypassing the graft and targeting the native coronary artery directly—is generally preferred, and both the 2018 ESC and 2021 ACC/AHA/SCAI guidelines support this approach with a Class IIa recommendation [62,63]. When native-vessel PCI is not anatomically feasible, repeat SVG PCI may be the only percutaneous option, and should be performed carefully with a low threshold for imaging.

### 9.4. Recurrent ISR

Recurrent ISR—defined as restenosis occurring within a segment that has already undergone one or more prior PCI interventions—is one of the most clinically challenging problems in contemporary interventional cardiology and warrants more detailed consideration than first-episode ISR.

The first priority in recurrent ISR is mechanistic reassessment. Repeat PCI without understanding why the previous treatment failed is likely to produce another failure. Intracoronary imaging is essential: IVUS or OCT will typically reveal whether recurrence is driven by inadequate expansion (suggesting that prior interventions did not resolve an underlying mechanical problem), progressive NIH (suggesting a biological substrate that was not adequately treated), neoatherosclerosis (particularly relevant in very late recurrences), multilayer stent complexity, or stent fracture.

Multilayer ISR—where multiple overlapping stent layers create dense scaffolding that impairs drug penetration, increases metallic density per vessel length, and complicates further expansion—represents the most difficult subset. Each additional stent layer reduces the likelihood of achieving adequate expansion and drug delivery, and the addition of yet another DES layer may perpetuate the cycle [96]. In this setting, strategies that avoid additional metal are particularly attractive, specifically DCB or VBT, when lesion preparation can achieve adequate lumen gain.

VBT retains a meaningful role in recurrent multilayer DES-ISR precisely because it delivers antiproliferative therapy without adding stent material [81]. VBT carries a Class IIb recommendation in the ACC/AHA for this indication, reflecting real but limited efficacy and a recognition that access and expertise are restricted [62]. Where VBT is unavailable or technically unsuitable, intensive lesion preparation followed by DCB is a reasonable alternative, though recurrence rates in truly multilayer disease remain substantially higher than in first-episode ISR [97].

Optimising modifiable risk factors is equally important. Suboptimal low-density lipoprotein (LDL) control, poorly controlled diabetes, and ongoing smoking are all associated with increased ISR recurrence and should be addressed alongside mechanical reintervention. Where risk factor modification has been inadequate, it should be optimised in parallel with any mechanical treatment decision.

For patients with truly refractory or recurrent ISR in whom repeated PCI has not provided durable benefit, Heart Team discussion should be prompted to evaluate the role of CABG [62,63,65]. The decision to refer for surgery requires balancing the procedural risk with the realistic expectation that further PCI will again fail.

### 9.5. Stent Fracture

Stent fracture should be suspected in any patient with ISR at a site of known tortuosity, hinge points (particularly the right coronary artery), or long overlapping stent segments [98]. Confirmation requires IVUS or OCT, as angiography alone frequently misses partial fractures or fails to precisely localise the discontinuity.

Treatment depends on the extent of structural disruption. Focal fractures with limited associated ISR and preserved stent integrity elsewhere may respond to lesion preparation followed by DCB or targeted repeat DES to bridge the fracture site. Complete or highly disrupted fractures, or those associated with severe restenosis, generally require repeat DES to restore both mechanical scaffolding and drug delivery. High-pressure non-compliant balloons, scoring balloons, or IVL may be needed to address calcium that contributed to the fracture before definitive device placement [98].

## 10. Future Directions

The field of ISR management is advancing on several parallel fronts, each with meaningful potential but also with important uncertainties that require prospective evidence before practice can change.

The development of sirolimus-based DCB platforms represents the most clinically immediate area of evolution. Paclitaxel-based DCBs have an established evidence base in ISR, but sirolimus’s distinct pharmacokinetic profile—slower absorption, broader intramural distribution—offers a theoretical advantage in tissue penetration and drug retention that may be relevant for biological ISR substrates.

The SELUTION SLR platform, which employs a biodegradable polymer matrix to sustain sirolimus release rather than relying on a single bolus transfer, has completed enrolment in the SELUTION4ISR RCT comparing it to standard of care. One-year results are encouraging, but the key question of whether sustained-release sirolimus DCB improves outcomes beyond what is achievable with current platforms at longer follow-up remains open [75]. Head-to-head comparisons between sirolimus-based and paclitaxel-based DCBs in the ISR setting—rather than separate comparisons against balloon angioplasty or DES—are needed to resolve which platform, if either, offers clinically meaningful superiority.

Optimal lesion preparation is a prerequisite for successful definitive ISR therapy, and improvements in preparation tools may reduce the proportion of cases where residual calcification, underexpansion, or suboptimal drug delivery limit outcomes. Second-generation IVL systems with refined pulse patterns may enable more controlled calcium modification with less risk of vessel trauma, particularly in heavily calcified ISR where conventional balloons fail and atherectomy risks perforation or burr entrapment. Refined orbital atherectomy and laser systems are also being evaluated in broader calcified coronary disease and may eventually accumulate sufficient ISR-specific data to support more confident recommendations. At present, however, all lesion preparation evidence in ISR derives from small observational series or data extrapolated from de novo calcified lesion trials, and randomised evidence in the ISR substrate specifically is lacking.

Artificial intelligence (AI)-assisted intracoronary imaging analysis represents a potential tool for improving the consistency and speed of ISR substrate characterisation. Automated quantification of stent expansion, NIH volume, and neoatherosclerotic tissue characterisation from IVUS and OCT pullbacks may reduce operator-dependent variability, improve reproducibility of ISR substrate identification, and support real-time treatment decision-making. Early proof-of-concept studies are promising, though prospective clinical validation in ISR-specific trials is required. This is an important distinction: better characterisation is a plausible pathway to better treatment, but it is not equivalent to demonstrated benefit.

The optimal medical management of patients with ISR remains incompletely defined. Several important questions are unanswered. First, whether DAPT duration should differ after repeat DES for ISR compared with de novo DES implantation has not been studied in a specifically designed trial; current practice extrapolates from de novo PCI data and small post hoc subgroup analyses. Second, whether intensive lipid-lowering with PCSK9 inhibitors or other novel agents reduces the biological ISR substrate—particularly neoatherosclerosis—beyond their established secondary prevention benefit is biologically plausible but unproven. Third, colchicine and other anti-inflammatory agents, which have now demonstrated mortality benefit in stable CAD (LoDoCo2) and post-ACS populations (COLCOT), have not been specifically evaluated in the ISR population, where their effect on NIH and recurrence risk is biologically credible but uninvestigated [99,100].

The most important gap in the current evidence base is the absence of large, ISR-specific randomised trials with hard clinical endpoints and long follow-up. Most existing RCTs use angiographic surrogates (percentage diameter stenosis, late lumen loss, binary restenosis at 6–12 months) as primary endpoints, and these do not reliably predict long-term cardiovascular outcomes in the ISR setting. Trials comparing imaging-guided to angiography-guided ISR management—where imaging guidance means not just performing IVUS or OCT, but acting on the findings to change the procedural strategy—are needed to demonstrate whether the additional cost and complexity of routine imaging translates into meaningful outcome benefit for patients. Similarly, trials specifically powered for ISR in high-risk subsets (recurrent ISR, multilayer ISR, LM-ISR, and CTO-ISR) are currently lacking, and the available subgroup data from broader coronary intervention trials are insufficiently powered to guide practice in these groups.

## 11. Conclusions

ISR remains a clinically relevant and mechanistically heterogeneous complication of coronary stenting, occurring across a spectrum from focal underexpansion to diffuse neoatherosclerosis and multilayer recurrent disease. Its management requires a substrate-first approach: identifying the dominant mechanism with intracoronary imaging before selecting lesion preparation strategy and definitive therapy.

Intracoronary imaging with IVUS or OCT is central to this approach and is supported by current international guidelines, though the evidence base is predominantly observational, and routine use must be balanced against local expertise, cost, and patient-level factors including contrast load. Coronary angiography with physiological assessment remains the initial diagnostic step, and imaging is most valuable when the mechanism of ISR is not angiographically apparent.

Lesion preparation with high-pressure balloon angioplasty, and adjunctive scoring balloons, IVL, or atheroablative therapy when calcification or resistance limits expansion, should precede definitive treatment. The choice between DCB and repeat DES should be individualised: DCB is preferred when avoiding additional metal layers is important—particularly in multilayer, recurrent, or small-vessel ISR, or in high bleeding-risk patients—while repeat DES is appropriate when mechanical failure requires scaffolding correction or when prior DCB therapy has not been durable.

The most important gaps in the current evidence are the absence of ISR-specific randomised trials powered for hard clinical endpoints, the lack of head-to-head DCB platform comparisons, and unresolved questions about optimal DAPT duration and the role of novel anti-inflammatory and lipid-lowering strategies in reducing ISR recurrence. Addressing these gaps will be essential for moving the field from expert consensus and surrogate-endpoint extrapolation toward practice grounded in patient-level outcome evidence.

## Figures and Tables

**Table 1:** A summary table for ISR risk factors. ACS, acute coronary syndrome; ISR, in-stent restenosis; SVG, saphenous vein graft. Adapted from Klein et al. [11].

Coronary In-Stent Restenosis Risk Factors		
**Patient factors**	**Anatomical factors**	**Procedural factors**
Female	Lesion length > 20 mm	Stent underexpansion
Old age	Diameter < 3 mm	Stent fracture
Smoking	Ostial lesion	Bare-metal stent
ACS presentation	Bifurcation lesion	Stenosis proximal and distal to stent
Hypertension, Dyslipidaemia	Multivessel lesion	Dissection involving media or >3 mm length
Diabetes mellitus	Chronic total occlusion	Multiple stent layers
Chronic kidney disease	Severe calcification	
Recurrent ISR	SVG graft	

**Table 2:** Comparison between the use of IVUS and OCT in coronary artery lesion and identifying ISR substrates. +++, excellent; ++, good; +, adequate; -, poor or not advised; IVUS, intravascular ultrasound; OCT, optical coherence tomography. Adapted from Klein et al. [11].

Application of IVUS and OCT for Lesion and ISR Assessment		
	**IVUS**	**OCT**
***Assessing LM lesion***	+++	+
***Assessing lesion characteristics***		
Thin cap fibroatheroma	-	+++
Thrombus	+	+++
Plaque rupture	++	+++
Calcified nodules	+	+++
Dissection	++	+++
Positive remodelling	+++	+
Plaque burden	+++	+
Aorto-ostial disease	+++	-
***Stent apposition or expansion***	++	+++
***Stent failure***		
Neointimal hyperplasia	+	++
Underexpansion or malapposition	++	+++
***Renal impairment***	+++	+

## Data Availability

No new data were created or analysed in this study. Data sharing is not applicable to this article.

## Abbreviations

ACC/AHA	American College of Cardiology/American Heart Association
BA	Balloon angioplasty
BMS	Bare-metal stents
CABG	Coronary artery bypass graft
CAD	Coronary artery disease
CTO	Chronic total occlusion
DAPT	Dual antiplatelet therapy
DCB	Drug-coated balloons
DES	Drug-eluting stents
ELCA	Excimer laser coronary atherectomy
ESC	European Society of Cardiology
FFR	Fractional flow reserve
ISR	In-stent restenosis
IVL	Intravascular lithotripsy
IVUS	Intravascular ultrasound
JCS/JSCVS	Japan Circulation Society/Japanese Society for Cardiovascular Surgery
LAD	Left anterior descending artery
LCx	Left circumflex artery
LM	Left main
MACE	Major adverse cardiac events
NIH	Neointimal hyperplasia
OCT	Optical coherence tomography
PCI	Percutaneous coronary intervention
RA	Rotational atherectomy
RCT	Randomised controlled trial(s)
SCAI	Society for Cardiovascular Angiography and Interventions
SVG	Saphenous vein graft
TLR	Target lesion revascularisation
TVF	Target vessel failure
VBT	Vascular brachytherapy
